# Reappraisal of *Pseudomonas aeruginosa* hospital-acquired pneumonia mortality in the era of metallo-β-lactamase-mediated multidrug resistance: a prospective observational study

**DOI:** 10.1186/cc5006

**Published:** 2006-08-01

**Authors:** Alexandre Prehn Zavascki, Afonso Luís Barth, Juliana Fernandez Fernandes, Ana Lúcia Didonet Moro, Ana Lúcia Saraiva Gonçalves, Luciano Zubaran Goldani

**Affiliations:** 1 Infectious Diseases Service, Hospital São Lucas da Pontifícia Universidade Católica do Rio Grande do Sul, Porto Alegre – RS, Brazil; 2 Medical Sciences Postgraduate Program, Universidade Federal do Rio Grande do Sul, Porto Alegre – RS, Brazil; 3 Microbiology Unit, Clinical Pathology Service, Hospital de Clínicas de Porto Alegre, Porto Alegre – RS, Brazil; 4 Division of Infectious Diseases, Hospital de Clínicas de Porto Alegre, Porto Alegre – RS, Brazil

## Abstract

**Introduction:**

Hospital-acquired pneumonia (HAP) due to *Pseudomonas aeruginosa *is associated with high mortality rates. The metallo-β-lactamases (MBLs) are emerging enzymes that hydrolyze virtually all β-lactams. We aimed to assess *P. aeruginosa *HAP mortality in a setting of high-rate MBL production

**Methods:**

A prospective cohort study was performed at two tertiary-care teaching hospitals. A logistic regression model was constructed to identify risk factors for 30-day mortality.

**Results:**

One-hundred and fifty patients with *P. aeruginosa *HAP were evaluated. The 30-day mortality was 37.3% (56 of 150): 57.1% (24 of 42) and 29.6% (32 of 108) for patients with HAP by MBL-producing *P. aeruginosa *and by non-MBL-producing *P. aeruginosa*, respectively (relative risk, 1.93; 95% confidence interval (CI), 1.30–2.85). The logistic regression model identified a higher Charlson comorbidity score (odds ratio, 1.21; 95% CI, 1.04–1.41), presentation with severe sepsis or septic shock (odds ratio, 3.17; 95% CI, 1.30–7.72), ventilator-associated pneumonia (odds ratio, 2.92; 95% CI, 1.18–7.21), and appropriate therapy (odds ratio, 0.24; 95% CI, 0.10–0.61) as independent factors for 30-day mortality. MBL production was not statistically significant in the final model.

**Conclusion:**

MBL-producing *P. aeruginosa *HAP resulted in higher mortality rates, particularly in patients with ventilator-associated pneumonia, most probably related to the less frequent institution of appropriate antimicrobial therapy. Therapeutic approaches should be reviewed at institutions with a high prevalence of MBL.

## Introduction

Hospital-acquired pneumonia (HAP), particularly ventilator-associated pneumonia (VAP), causes considerable morbidity and mortality despite antimicrobial therapy and advances in supportive care [1,2]. It is the second most frequent nosocomial infection and is the major cause of death among hospital-acquired infections [1]. *Pseudomonas aeruginosa *is a leading cause of nosocomial infections all over the world, especially of HAP and VAP, when it usually ranks as the first or second causative pathogen [1-3]. This organism is uniquely problematic because of a combination of inherent resistance to many drug classes and its ability to acquire resistance to all relevant treatments [3]. Severe infections due to *P. aeruginosa *are associated with high mortality regardless of appropriate antimicrobial therapy [3].

The metallo-β-lactamases (MBLs) have recently emerged as one of the most worrisome resistance mechanisms owing to their capacity to hydrolyze, with the exception of aztreonam, all β-lactam agents, including the carbapenems; and also because their genes are carried on highly mobile elements, allowing easy dissemination of such genes among Gram-negative rods [4]. MBLs have been rapidly spreading through many countries, particularly from Southeast Asia, Europe, and Latin America [4-6]. The emergence of these enzymes drastically compromises effective treatments of nosocomial infections by this organism, bringing us closer to the much feared 'end of antibiotics' [4-6].

We have recently demonstrated that nosocomial infections due to metallo-β-lactamase-producing *P. aeruginosa *(MBL-PA) isolates have been associated with higher mortality rates [7]. In the present article, we aimed to assess the mortality of the subset of patients with HAP due to *P. aeruginosa *in a setting of high-rate MBL production.

## Materials and methods

### Study design and patients

A contemporary cohort study of consecutive patients with *P. aeruginosa *nosocomial infections was performed at two tertiary-care teaching hospitals in Porto Alegre, southern Brazil. The study period was from September 2004 to June 2005 at São Lucas Hospital, a 600-bed hospital, and from January to June 2005 at Hospital de Clínicas de Porto Alegre, a 1,200-bed hospital [7].

In the current study, we analyzed patients ≥ 18 years, who did not have cystic fibrosis, who had been diagnosed with HAP defined as follows. First, the presence of positive cultures for *P. aeruginosa *either recovered from respiratory secretions (>10^6 ^cfu/ml from endotracheal aspirates or >10^4 ^cfu/ml from bronchoalveolar lavage) after 48 hours of hospital admission, or within 48 hours if the patient had been hospitalized in the past 60 days, or recovered from blood without the presence of any other pathogen in respiratory secretions. Second, the presence of a radiographic infiltrate that was new or progressive, along with the presence of two or more of the following criteria: fever (temperature >38°C) or hypothermia (temperature <36°C), purulent sputum, leukocytosis (>10,000 cells/mm^3^) or leukopenia (<4,000 cells/mm^3^), and a decline in oxygenation. Sputum was considered purulent if >25 neutrophiles and <10 epithelial cells per high power field were present. VAP was defined as HAP that developed after 48 hours of mechanical ventilation.

Patients were excluded if they did not fulfill these criteria for HAP. Patients were followed from the first isolation of *P. aeruginosa *to discharge from hospital or to death. Antimicrobial agents used were at the discretion of the patient's physicians, not the investigators. The ethics review boards of both hospitals approved the study.

### Data collection

Data were collected from medical charts and/or hospital computer system databases, both during and after the patients' hospitalization. The researchers were blinded for the MBL status of *P. aeruginosa *isolates during data collection.

### Microbiology

Conventional microbiology methods were used for *P. aeruginosa *identification, and susceptibility tests were performed by disk-diffusion methods according to Clinical and Laboratory Standards Institute, (formerly National Committee for Clinical Laboratory Standards), guidelines [8]. Susceptibility was tested for amikacin, aztreonam, cefepime, ceftazidime, ciprofloxacin, imipenem, meropenem, piperacillin-tazobactam, and polymyxin B. Susceptibility of the latter was determined using the interpretative criteria (≥ 14 mm) proposed elsewhere [9]. All isolates resistant to ceftazidime were screened for MBL production with ceftazidime in the presence of 3 μl 2-mercaptopropionic acid as previously described [10].

### Variables and definitions

The main outcome was 30-day mortality. Other secondary outcomes were the length of need for vasoactive drugs and the length of mechanical ventilation (both were assessed in survivors).

The variable in the study was MBL production. Other independent variables analyzed included the following: age; sex; Charlson comorbidity score [11] (assessed at the moment of HAP diagnosis); baseline diseases; iatrogenic immunosuppression, such as chemotherapy-induced neutropenia (neutrophile count ≤ 1,000 mm^3^), and/or receipt of corticoid drugs (prednisone ≥ 10 mg daily or equivalent doses) or other immunosuppressive agents for >14 days; the presence of other concomitant infections (infections by other organisms at a site other than the lung, excluding coagulase-negative staphylococci in a single blood culture); a previous surgical procedure during the hospital stay; the length of hospital stay (before the diagnosis of HAP); presentation of HAP with severe sepsis or septic shock [12]; infection by *P. aeruginosa *at more than one site (not including patients with HAP and bacteremia); polymicrobial infection (isolation of another organism from the respiratory secretions at the moment of *P. aeruginosa *HAP diagnosis); associated bacteremia (isolation of *P. aeruginosa *from one or more blood samples); VAP; receiving appropriate empirical therapy (defined as the administration of an antimicrobial agent to which the isolate was susceptible *in vitro *in ≤ 24 hours of sample collection); receiving appropriate definitive therapy (defined as the use for at least 48 hours of an antimicrobial agent to which the isolate was susceptible *in vitro*); time to receiving appropriate definitive therapy (only for those who have not received appropriate empirical therapy; time in days from the sample collection to the first dose of appropriate therapy); and combination antibiotic treatment (treatment with more that one agent with *in vitro *susceptibility).

Aminoglycosides in monotherapy were not considered appropriate treatment therapy despite *in vitro *susceptibility [3].

### Statistical analysis

All statistical analyses were carried out using SPSS for Windows, version 13.0. The relative risk (RR) and the 95% confidence interval (CI) were calculated for 30-day mortality of patients with MBL-PA HAP and of patients with non-MBL-PA HAP. *P *values were calculated using the chi-squared test or Fischer exact test for categorical variables, and using Student's *t *test or the Wilcoxon rank-sum test for continuous variables.

A logistic regression model was constructed to identify independent factors associated with 30-day mortality using a forward stepwise approach. Variables for which the *P *value was < 0.20 in univariate analysis were included in the model. *P *= 0.05 was set as the limit for acceptance or removal of the terms in the model. MBL production remained in the model independent of the *P *value. All tests were two-tailed and *P *≤ 0.05 was considered significant.

## Results

### Patients and mortality

A total of 473 patients presented the isolation of *P. aeruginosa *after >48 hours of hospital admission. Of these, 171 presented the isolation of *P. aeruginosa *in respiratory secretions. Twenty-one patients were excluded because they did not fulfill the criteria for HAP. A total of 150 patients were analyzed. Forty-two (28.0%) patients presented MBL-PA HAP.

The 30-day mortality was 37.3% (56 of 150) and represented 76.7% of the 73 deaths. Among patients with MBL-PA HAP the 30-day mortality was 57.1% (24 of 42), compared with 29.6% (32 of 108) for non-MBL-PA patients (RR, 1.93; 95% CI, 1.30–2.85; *P *< 0.01). The overall mortality rate was 18.7 per 1,000 patient-days: 26.5 per 1,000 patient-days among MBL-PA-infected patients and 15.8 per 1,000 patient-days among non-MBL-PA-infected patients (*P *= 0.02). The median length of follow-up was 19 days (interquartile range, 9–31 days): 16 days (interquartile range, 7–28 days) for those patients with MBL-PA HAP and 19.5 days (interquartile range, 10–31.5 days) for those patients with non-MBL-PA HAP (*P *= 0.19). The median length of follow-up of those patients who did not die within 30 days did not differ between patients with MBL-PA HAP and patients with non-MBL-PA HAP (29.5 days (interquartile range, 22–71 days) versus 24.5 days (interquartile range, 14–39.5 days), respectively; *P *= 0.22).

Fifty-five patients (36.7%) had VAP. Patients with VAP had a 30-day mortality of 60.0% (33 of 55): 77.3% (17 of 22) for patients with MBL-PA VAP compared with 48.5% (16 of 33) for those patients with non-MBL-PA VAP (RR, 1.59; 95% CI, 1.05–2.42; *P *= 0.03).

### Risk factors for mortality

The characteristics of patients according to 30-day mortality are presented in Table 1. Factors associated with mortality within 30 days in the univariate analysis were severe sepsis or septic shock, VAP, and bacteremia. Comorbidity scores were higher in patients who died within 30 days, but there was no statistically significant difference. Both empirically appropriate therapy and receiving appropriate therapy at any moment were significant protective factors for 30-day mortality; however, a greater effect was observed for therapy at any time.

Considering only patients who received appropriate therapy (*n *= 109), there was no statistically significant difference in mortality rates according to the time to administration of appropriate therapy. The 30-day mortality rates were 21.7% (≤ 24 hours), 32.0% (>24 hours but ≤ 72 hours), and 34.2% (>72 hours) (*P *= 0.41).

### Multivariate analysis

The results of multivariate analysis are presented in Table 2. The Charlson score, severe sepsis or septic shock, VAP, and appropriate treatment at any moment were significantly associated with 30-day mortality. Bacteremia and antimicrobial combination were not statistically significant and were excluded from the model. MBL production was not significantly associated with the outcome in the final model, but was statistically significant in the multivariate model (RR, 2.84; 95% CI, 1.24–6.52, *P *= 0.01) before the inclusion of appropriate antimicrobial therapy in the model. Specific comorbidities such as cirrhosis and AIDS were not included in the model because they were significantly associated with higher Charlson scores (data not shown).

### Secondary outcomes

Among the 77 survivors, patients with MBL-PA HAP presented significantly longer length of need for vasoactive drug therapy than non-MBL-PA patients (mean, 17.5 ± 3.5 days versus 3.6 ± 3.3 days; *P *< 0.001). The length of mechanical ventilation was also longer for patients with MBL-PA HAP than for non-MBL-PA patients, although without statistical significance (mean, 13.0 ± 9.0 days versus 7.8 ± 4.9 days; *P *= 0.12).

### Resistance patterns

A total of 38 distinct antibiotic resistance profiles were observed in *P. aeruginosa *isolates, but only six distinct patterns were observed among MBL-PA isolates. These latter profiles are presented in Table 3. Among non-MBL-PA isolates, 36 resistance profiles were found. The commonest profile was susceptibility to all tested drugs (27 patients, 25.0%), followed by susceptibility to all drugs except aztreonam (14 patients, 13.0%), and susceptibility to ceftazidime and piperacillin-tazobactam and resistance to the other drugs (10 patients, 9.3%). Other profiles each accounted for 6.5% or less of the total.

### MBL-producing *P. aeruginosa *HAP

Among the 42 patients with MBL-PA HAP, 41 received antimicrobial therapy and one did not receive any antibiotic. This latter patient had a missed diagnosis of HAP and died after four days of the onset of infection. Twenty-one (51.2%) of the 41 treated patients received appropriate therapy: 10 patients (47.6%) received such therapy in ≤ 72 hours, and three patients (14.3%) received appropriate antibiotic in ≤ 24 hours. Patients who received any therapy in ≤ 72 hours (*n *= 10) tended to present a lower 30-day mortality than those who received therapy after 72 hours (*n *= 31): 30.0% and 64.5%, respectively (RR, 0.45; 95% CI, 0.17–1.24; *P *= 0.06). Antimicrobial therapies of patients with MBL-PA HAP are presented in Table 4.

Considering only those 21 patients who received appropriate therapy, there was no statistically significant difference in mortality between receiving therapy in ≤ 72 hours (*n *= 9) and in >72 hours (*n *= 12) for 30-day mortality (33.3% versus 50.0%; RR, 0.67; 95% CI, 0.23–1.97; *P *= 0.38). Among these patients, no specific antibiotic agent was associated with lower mortality (*P *= 0.54).

## Discussion

MBL production is an emerging resistance mechanism in Gram-negative rods, particularly in *P. aeruginosa *[5,6]. In a recent article we showed that nosocomial infections due to MBL-PA were associated with increased mortality when compared with those infections caused by non-MBL-PA isolates, confirming that such a resistance mechanism is actually a clinical threat [7]. In this latter study, all sites of nosocomial infections were analyzed together but no data were available regarding clinical outcomes of more severe infections, such as HAP. The current study was carried out in order to reappraise the mortality of HAP, which is usually associated with high mortality rates, especially among critically ill patients [1,2], in a setting of high prevalence of MBL production. To the best of our knowledge, this was the first study to assess the impact of this emerging resistance mechanism on the outcome of patients with HAP.

Our study showed a high mortality in patients with HAP by *P. aeruginosa*, and MBL production by these isolates significantly increased the mortality of these patients. This effect was probably mediated by a more frequent inappropriateness of antimicrobial therapy for MBL-PA infections, considering that MBL production was not significantly associated with 30-day mortality when the variable administration of appropriate therapy was included in the multivariate analysis.

Both presentation with severe sepsis or septic shock and VAP had the strongest impact on 30-day mortality, supporting the importance of these factors in overall mortality as recognized in many studies [13-15]. Higher comorbidity scores had also a significant impact on the outcome of patients.

Our study did not demonstrate a significant effect of early appropriate therapy on mortality. Actually, a significant effect was not shown even adjusting for the comorbidity score, presentation of severe sepsis or septic shock, and VAP in patients who had received appropriate therapy (data not shown). This might be caused by the fact that most of patients who received appropriate therapy (66.1%, 72 of 109) received it in <72 hours; it may therefore be possible that our sample size lacks sufficient power to detect differences within this period of time. Nevertheless, crude analysis of mortality among patients who received appropriate therapy showed, although without statistical significance, lower mortality rates for those who started treatment earlier, particularly within 72 hours. Since early therapy is recognizably associated with better outcomes [2,14,15], we emphasize its importance and attribute, at least partially, the lack of statistical significance in our multivariate model to the reason exposed earlier.

Few antibiotic resistance profiles were observed among MBL-PA HAP patients. Four isolates were unexpectedly susceptible to piperacillin-tazobactam. Such an interesting finding, however, has already been reported previously [16,17]. MBLs have been a major determinant of carbapenem resistance at our institutions [18]. However, other resistance mechanisms to these agents were also present in some isolates, such as the loss of OprD outer membrane protein in the case of imipenem, and this latter mechanism with an associated overexpression of the MexAB-OprM efflux pump, as is the case for meropenem [18,19].

Worrisome high mortality rates were observed among patients with MBL-PA HAP despite appropriate therapy, particularly among those with VAP. Although no specific antibiotic proved to be significantly associated with lower mortality, aztreonam in monotherapy presented the lowest mortality among appropriate treatments for MBL-PA HAP (two of eight patients, 25.0%). All patients with VAP who were treated with this antibiotic in monotherapy died, however. Nevertheless, owing to the relatively small sample size, no definitive conclusion about superiority of any antibiotic for treatment of MBL-PA HAP can be made.

A limitation of our study was that patients who were discharged within 30 days were not followed-up after their hospitalizations, and it is possible that some of them could have died after hospital discharge within this period. This potential bias might not have influenced our results, however, since the lengths of follow-up of patients who have not presented the outcome did not differ between MBL-PA patients and non-MBL-PA patients.

Although it was not the scope of this study to investigate the molecular epidemiology of MBL-PA isolates, horizontal dissemination of these isolates has been demonstrated in these institutions, with SPM-1 being the most common MBL type [7,19].

## Conclusion

MBL production by *P. aeruginosa *determined a significant increase in mortality of patients with HAP, particularly of patients with VAP. A better therapeutic approach is required to improve outcomes of patients with MBL-PA HAP. Other investigations to determine the optimal treatment for these infections are required.

## Key messages

• 	High mortality rates were observed for patients with HAP, particularly VAP, due to *P. aeruginosa*.

• 	MBL production by *P. aeruginosa *significantly increases mortality rates of patients with HAP.

• 	The effect of MBL production on mortality was probably mediated by a more frequent inappropriateness of antimicrobial therapy for infections due to *P. aeruginosa *producing this enzyme.

• 	Presentation with severe sepsis or septic shock, VAP, higher comorbidity score, and inappropriateness of treatment were independently associated with the 30-day mortality.

• 	The optimal treatment for infections due to MBL-PA should be further investigated.

## Abbreviations

CI = confidence interval; HAP = hospital-acquired pneumonia; MBL = metallo-β-lactamase; MBL-PA = metallo-β-lactamase-producing *Pseudomonas aeruginosa*; RR = relative risk; VAP = ventilator-associated pneumonia.

## Competing interests

This study received financial support from Coordenação de Aperfeiçoamento de Pessoal de Nível Superior – CAPES, Ministry of Education, Brazil, and from Fundação de Incentivo a Pesquisa e Eventos – FIPE, Hospital de Clínicas de Porto Alegre. The study sponsor had no role in the study design, data collection, data analysis, data interpretation, or writing the report. The authors disclose no potential conflict of interest.

## Authors' contributions

APZ, ALB and LZG conceived the study. APZ wrote the first draft of the report. All authors contributed to the final draft. APZ performed the analysis, and ALB and LZG contributed to data interpretation. ALSG carried out microbiology tests and prepared the data for analysis. ALDM and JFF carried out the cohort follow-up, and extracted and prepared the data for analysis.

## Figures and Tables

**Table 1:** Characteristics of patients according to 30-day mortality

	30-day mortality		
	
Variable	Yes (*n *= 56)	No (*n *= 94)	*P*
Age (years)	66.4 ± 18.4	60.4 ± 17.9	0.38
Sex (male)	42 (75.0)	63 (67.0)	0.40
Charlson score	4 (2–6)	3 (2–6)	0.15
Comorbidities			
Neurological	14 (48.3)	44 (36.4)	0.33
Cardiac	20 (69.0)	69 (57.0)	0.33
Pulmonary	12 (41.4)	55 (45.5)	0.85
Malignancy	6 (20.7)	21 (17.4)	0.88
Diabetes	10 (34.5)	29 (24.0)	0.36
Renal	4 (13.8)	28 (23.1)	0.20
Cirrhosis	4 (13.8)	12 (9.9)	0.37
AIDS	2 (6.9)	8 (6.6)	0.61
Immunosuppression	23 (41.1)	33 (35.5)	0.58
Other infections	17 (30.4)	25 (26.6)	0.76
Previous surgery	24 (42.9)	38 (40.4)	0.90
Length of hospital stay (days)	16.5 (7.5–30)	15.5 (5–30)	0.52
Severe sepsis or septic shock	40 (71.4)	31 (33.0)	<0.001
Ventilator-associated pneumonia	33 (58.9)	22 (23.4)	<0.001
>1 site	7 (12.5)	15 (16.0)	0.73
Polymicrobial pneumonia	23 (41.1)	30 (31.9)	0.38
Bacteremia	12 (21.4)	9 (9.6)	<0.05
Appropriate therapy			
At any moment	31 (55.5)	78 (83.0)	<0.001
≤ 24 hours	11 (19.6)	35 (37.2)	<0.05
Time to initiate appropriate therapy (days)	4.5 ± 2.1	5.1 ± 5.1	0.60
Combination therapy (*n *= 109)	3 (9.7)	15 (19.9)	0.18

Data presented as the mean ± standard deviation, as the median (interquartile range), or as *n *(%).

**Table 2:** Multivariate analysis of factors associated with 30-day mortality Only variables of the final model are presented.

	30-day mortality	
	
Variable	Odds ratio (95% confidence interval)	*P*
Metallo-β-lactamase	1.77 (0.72–4.36)	0.21
Charlson	1.21 (1.04–1.41)	0.02
Severe sepsis or septic shock	3.17 (1.30–7.72)	0.01
Ventilator-associated pneumonia	2.92 (1.18–7.21)	0.02
Appropriate antimicrobial therapy	0.24 (0.10–0.61)	<0.01

**Table 3:** Antibiotic resistance profiles of 42 metallo-β-lactamase-producing *Pseudomonas aeruginosa*

Profile	Antibiotic							*n*	%
			
	Polymyxin B	Aztreonam	Piperacillin-tazobactam	Amikacin	Ciprofloxacin	Ceftazidime	Cefepime		
1	Susceptible	Susceptible	Resistant	Resistant	Resistant	Resistant	Resistant	17	40.5
2	Susceptible	Resistant	Resistant	Resistant	Resistant	Resistant	Resistant	14	33.3
3	Susceptible	Susceptible	Resistant	Susceptible	Resistant	Resistant	Resistant	6	14.8
4	Susceptible	Susceptible	Susceptible	Resistant	Resistant	Resistant	Resistant	2	4.8
5	Susceptible	Resistant	Susceptible	Resistant	Resistant	Resistant	Resistant	2	4.8
6	Susceptible	Resistant	Resistant	Resistant	Susceptible	Resistant	Resistant	1	2.4

**Table 4:** Therapy and mortality of patients with *Pseudomonas aeruginosa *producing metallo-β-lactamase hospital-acquired pneumonia

Treatment	Hospital-acquired pneumonia (*n *= 42)		Ventilator-associated pneumonia (*n *= 22)	
	
	Treated patients	30-day mortality (*n *= 24)	Treated patients	30-day mortality (*n *= 17)
Appropriate monotherapy	18 (42.9)	9 (50.0)	12 (54.5)	9 (75.0)
Aztreonam^a^	8	2	3	2
Polymyxin B^b^	6	4	5	4
Piperacillin-tazobactam^c^	4	3	4	3
Appropriate combination therapy	3 (7.1)	0 (0.0)	1 (4.5)	0 (0.0)
Polymyxin B + aztreonam	2	0	1	0
Aztreonam + amikacin	1	0	-	-
Nonappropriate combination therapy	3 (7.1)	2 (66.7)	1 (4.5)	0
Aztreonam + ceftazidime + amikacin	1	1	-	-
Imipenem + ceftazidime	1	0	1	0
Imipenem + ciprofloxacin	1	1	-	-
Nonappropriate monotherapy	17 (40.5)	12 (70.6)	8 (36.4)	8 (100)
Cefepime	7	5	3	3
Meropenem	6	3	2	2
Imipenem	2	2	2	2
Ceftazidime	1	1	1	1
Amikacin	1	1	-	-
Without therapy	1 (2.4)	1 (100)	-	-

^a^The association of *in vitro *nonsusceptible antibiotics were used in three patients: ceftazidime (one patient), cefepime (one patient), and ceftazidime + amikacin (one patient); all were survivors. ^b^One patient received the association of cefepime (*in vitro *nonsusceptible); survivor. ^c^One patient received the association of ciprofloxacin (*in vitro *nonsusceptible); nonsurvivor.
